# Analysis and Adjustment of Positioning Error of PSD System for Mobile SOF-FTIR

**DOI:** 10.3390/s19235081

**Published:** 2019-11-21

**Authors:** Liguo Qu, Jianguo Liu, Yasong Deng, Liang Xu, Kai Hu, Weifeng Yang, Ling Jin, Xiaoxiao Cheng

**Affiliations:** 1 Key Laboratory of Environmental Optics and Technology, Anhui Institute of Optics and Fine Mechanics, Chinese Academy of Sciences, Hefei 230031, China; lgqu@aiofm.ac.cn (L.Q.); jgliu@aiofm.ac.cn (J.L.);; 2 Science Island Branch of Graduate School, University of Science and Technology of China, Hefei 230026, China; 3 Key Laboratory of Optical Monitoring Technology for Environment, Anhui Province, Hefei 230031, China; 4 School of Physics and Electronic Information, Anhui Normal University, Wuhu 241000, China

**Keywords:** position sensitive detector, genetic algorithm, SOF-FTIR, sensor calibration

## Abstract

A PSD-based solar spot position detection system is developed for solar tracking closed-loop control of mobile SOF-FTIR (Solar Occultation Flux method based on Fourier Transform Infrared spectrometer). The positioning error factors of PSD (position sensitive detector) are analyzed in detail. A voltage model for PSD signal conditioning circuit has been established to investigate the noise factors. The model shows that the positioning error is mainly related to PSD dark current and circuit gain. A static voltage deduction calibration method based on genetic algorithm is proposed to eliminate the effect of dark current. The gain ratio between channels is calculated based on the fitting curve slope of discrete position data of PSD center point with different light intensity for circuit gain calibration. The positioning accuracy and precision are greatly enhanced, especially when the light intensity is weak, compared with uncalibrated results. The positioning accuracy of center, middle and edge areas of PSD can reach 0.14%, 0.49%, and 1.09%, respectively, after correction in the range of light intensity voltage from 40 mV to 20 V. The corresponding standard deviations of each region are 0.005, 0.009, and 0.014, respectively. The adjustment methods proposed in this paper improve both measurement accuracy and detection limit. The results demonstrate that the calibrated PSD positioning accuracy can meet the requirements of SOF-FTIR for solar tracking.

## 1. Introduction

SOF-FTIR (Solar Occultation Flux method based on Fourier Transform Infrared spectrometer) method is an optical remote sensing technique for quantifying fugitive VOC emissions from industrial sources. The FTIR is connected to the mobile solar tracker, which reflects the sunlight to the spectrometer. The SOF method can directly measure the wide band absorption spectroscopy of infrared sunlight, as well as the concentration of a gas column integrated along a straight line or surface. Combined with wind measurements, SOF-FTIR has been shown to be very useful to constrain emission of trace gases from source regions [1]. The SOF method requires very high accuracy in solar tracking. For example, an error smaller than 0.05% in tracking the total gas column is desirable for ground-based solar absorption FTIR to achieve a total CO_2_ column precision of 0.1% [2,3]. If one wants to maintain this for a tropospheric gas up to a solar zenith angle of 80°, a tracking accuracy of about 19 arc sec is required [2]. The instrument is placed in a vehicle which is moved across the plume by 60–100 km per hour to obtain the gas emission from a source. The light intensity will change greatly due to cirrus, haze, automobile turning or shade of trees, etc., while the car is moving. In this case, mobile SOF-FTIR is required to track the sun accurately and quickly during movement to satisfy the request of spectral resolution and signal-to-noise ratio. It is a typical high dynamic control system.

Previously, these major efforts were taken to improve the spectrometer itself, but the tracking quality also has to be considered for SOF-FTIR, which is still a key issue. The traditional solar tracking method is mainly based on the camera system to obtain the solar image, and then use image processing technology to extract the solar center point for solar tracking [4,5,6,7]. However, these methods can only form clear images when the irradiance of the sun is strong enough, and they are easily affected by weather conditions or geographical factors. Moreover, the slow scanning speed of camera and complex image processing algorithm are not suitable for real-time fast-moving solar tracking system.

Fortunately, the position sensitive detector (PSD) provides us with another method of spot location detection. PSD is an analogue optical sensor which consists of a special monolithic PIN photodiode with several electrodes in order to achieve detection in one or two dimensional position detection [8]. The output of the PSD is a function of the center of gravity of the total light quantity distribution on the active area. PSD has nanoscale position resolution and nanosecond time resolution when measuring the center of gravity of light spots. PSD has a series of advantages over traditional cameras, including fast response time, good positioning accuracy, and simple signal conditioning circuit [9,10]. Therefore, PSD has been widely used in a variety of applications, including micro/nano position measurement, vibration detection, tool alignment, aiming and guidance systems, etc., which use lasers or other light sources to directly illuminate the PSD surface for position detection [9].

Currently, the main challenge of PSD application in different fields is their positioning accuracy, which is mainly affected by circuit noise, tolerances of optical and electronic components, temperature drift of components, the dark current and environment stray light [11,12]. These factors seriously affect the resolution and accuracy of the PSD positioning system, which cannot be completely eliminated in the implementation process of the PSD system [13]. Therefore, many early researches have been carried out on these noise sources and their correction methods [11,12,13,14,15]. Study [11] analyzes the factors that generate substantial errors in the PSD’s response, which are divided into random errors produced by external interference, quantization noise errors produced by A/D conversion, and systematic errors caused by component tolerance and temperature change. The systematic error is source of considerable error that cannot be ignored and must be eliminated. Random errors, which are negligible compared with systematic errors, cannot be eliminated by calibration [11].

To improve the accuracy of a PSD system, some improved methods have been proposed from the perspective of algorithms or devices. In [11], the output voltage of four channels of PSD is collected when the infrared light source uniformly illuminates the PSD surface, and then the gain ratio of different channels is fitted by quadratic polynomial mathematical model to correct the circuit gain. This is an effective method to eliminate the effect of amplifying gain imbalance caused by circuit element tolerance. However, it cannot eliminate the effects of dark current and circuit noise, especially when the light source intensity is weak. In [12], a Kalman filter for the PSD system with laser source is firstly designed for recursively estimating the laser spot position value by linearly modeled from a series of measurements mixed with noises. To further implement the Kalman filter, the system needs to be modeled as accurate as possible. Obviously, this method can roughly correct the position estimation using the detection results, but the system’s measurement errors cannot be calibrated by this method.

The modulation of light source is used to avoid the effects of background light on the premise of stable light intensity [16,17]. In [16], the light intensity of laser diode is modulated with square-wave. The output of PSD can be filtered by a bandpass filter to obtain high SNR and high precision. In [17], Sinusoidal light intensity modulation and band-pass filtering technology are used to overcome the interference of background light and greatly improve the signal-to-noise ratio (S/N) of position detection system. However, such systems become unfeasible in many normal practical applications due to the need to be compatible with simpler light sources, reduce technical complexity, and reduce system cost [18]. Thus, a lot of position measurement systems based on PSD with continuous light sources are still produced and utilized in industrial production [18].

In this area of study, various methods have been adopted to correct distortion. In all cases, Laser Diode or laser-like light sources are often used as matching light source for PSD. The laser diode adapts well to the response wavelength of the PSD because of the high output power and good directivity. The PSD systems constructed with laser diode have better robustness than the continuously changing light source. In this paper, a PSD-based solar spot position detection system is developed for solar tracking closed-loop control of mobile SOF-FTIR. Sunlight is used as matching light source of PSD in the SOF-FTIR system. PSD is required to detect the solar altitude Angle accurately and quickly in dynamic light intensity variation in order to achieve high dynamics and precise loop control. However, the PSD 4-channel gain is always different due to asymmetric circuit parameters, which will introduce PSD incident spot positioning calculation error. In addition, the other major contributor to the positioning error is static output voltage, which is mainly caused by thermal noise, shot noise, and dark current of PSD, especially when the incident light intensity weakens. The purpose of this study is to solve these two problems.

## 2. Measurement Model and Methods

**Figure 1:**
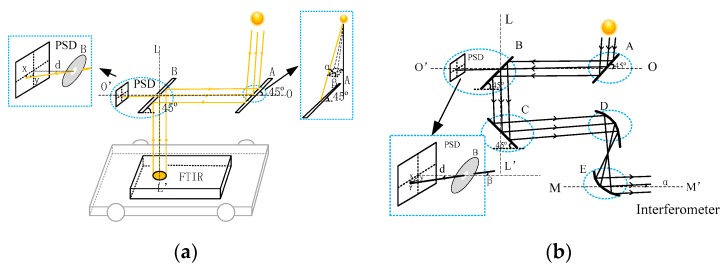
(**a**) The schematic diagram of SOF-FTIR system. (**b**) The external light path of FTIR.

The method adopted in this study is to use PSD to detect the position deviation of the incident point, and then input the PSD signal into the control loop of the tracker for servo control to conduct solar tracking. The schematic diagram of SOF-FTIR is shown in Figure 1a. The basic structure consists of FTIR spectrometer mounted on a moving vehicle and solar auto-tracking system based on PSD. The latter is an optical system that tracks the sun and reflects the light into the spectrometer independent of its position. The mirrors (A and B) are mounted in parallel. They are at a 45 degree-angle to the OO’ axis and can rotate synchronously along the LL’ axis. Mirror A can rotate along the OO’axis compared with mirror B. Light beam can reach the PSD surface by opening a hole with ∅0.5 mm in the center of the mirror B. Subsequently, we can drive mirrors A and B for tracking sun based on X-Y position, which is the point of incidence beam on the PSD surface calculated by the PSD output signal.

The schematic diagram of external light path of FTIR is shown in Figure 1b. The parameters of FTIR resolution and maximum wave number are as follows, $\Delta v=0.5{\;\mathrm{cm}}^{-1}$,${\;v}_{\max}=12,500{\;\mathrm{cm}}^{-1}$. The maximum divergence Angle $\alpha_{\max}$ of the beam, entering the interferometer after collimated by the parabolic reflector E, is 6.32 mrad according to the formula $\alpha_{\max}={\left(\Delta v/v_{\max}\right)}^{1/2}$. It is also known that the focal length of parabolic reflectors D and E is 152.4 mm and 100 mm, respectively. The maximum divergence angle of the incident beam to parabolic reflector D is 4.147 mrad (0.2376°) according to the imaging properties of the optical system. The maximum divergence angle β of the solar beam is also 4.147 mrad because the divergence angle of the parallel beam is not affected by the reflection of the plane mirror. According to the angle relation γ = d/tanβ (d = 40 cm), the increment γ of PSD spot displacement is 1.652 mm, which is the maximum acceptable solar tracking error of the system.

**Figure 2:**
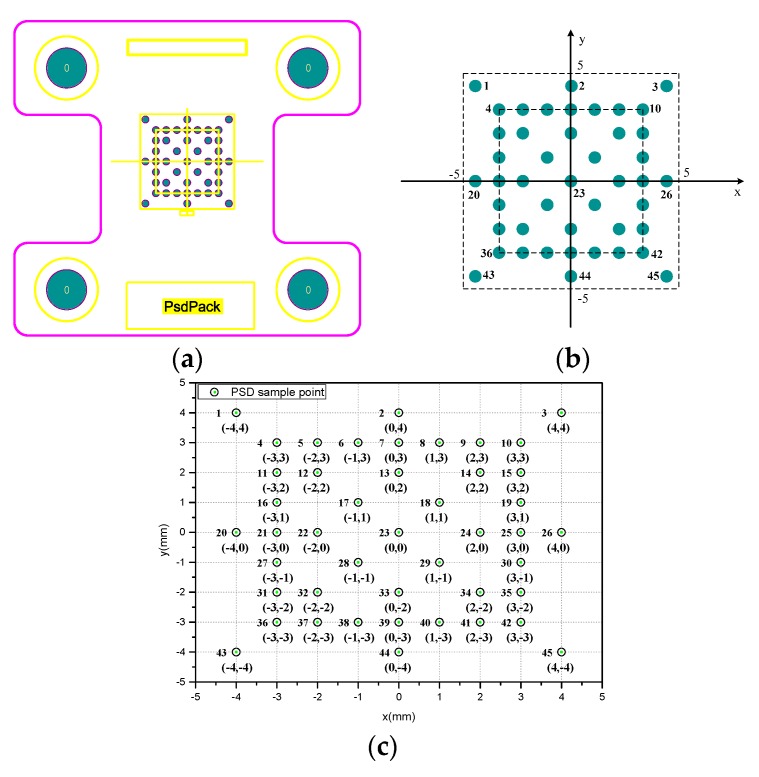
(**a**) The template with 45 0.5 mm diameter holes. (**b**) Template hole position coordinates. (**c**) Fixed coordinate points selected on the PSD (Number 1 through 45 from left to right and top to bottom).

As shown in Figure 2a,b, a template with forty-five 0.5 mm diameter holes is designed to verify the accuracy of spot positioning detection. These holes are distributed on the template evenly. The template and PSD are installed in parallel with a distance of 1 mm, and four sides are exactly aligned through location pins. A power-adjustable laser mounted on a precision three-Axis displacement platform emits parallel beams that shoot directly into the PSD through a template hole. The wavelength of the laser is 600 nm and the spot diameter is ∅800 μm. At different positions, a set of data of each point is acquired continuously by regulating laser power from weak to strong until output voltage saturation. Data is collected and stored in a three-dimensional array DATA[i][j][m] (i = {1,⋯,45}, j = {1,⋯,4}, m = {1,⋯,90}). Here, i represents sample point label, j represents output channel label of each sample point, and m represents the output data number of each channel at different light intensity. This means that 90 pieces of data are collected at per channel at each point. Nine sample points (label i = {1,2,3,20,23,26,43,44,45}) were selected as calibration points on the PSD. Their coordinates are (–4,4),(0,4),(4,4),(–4,0),(0,0),(4,0),(–4,–4),(0,–4),(4,–4), respectively. Other points are used to verify the validity of the calibration method.

**Figure 3:**
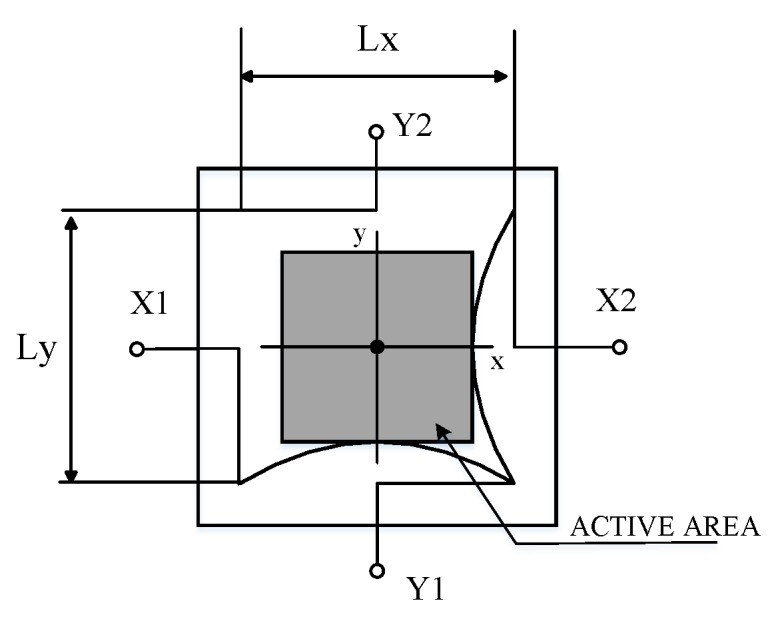
The active area chart of pin-cushion type PSD.

The PSD unit model used in the study is a commercial Hamamatsu S5990-01 with a two dimensional square $9\times 9{\;\mathrm{mm}}^{2}$ sensing area. The photocurrent from four electrodes are divided into 4 parts through the same resistive layer and extracted as position signals. As shown in Figure 3, the incidence spot position of pin-cushion type PSD are calculated by Equations (1) and (2) [9].

(1) $$\frac{\left(\mathrm{Ix}2\;+\;\mathrm{Iy}1\right)\;-\;\left(\mathrm{Ix}1\;+\;\mathrm{Iy}2\right)}{\mathrm{Io}}\;=\;\frac{2x}{\mathrm{Lx}}$$

(2) $$\frac{\left(\mathrm{Ix}2\;+\;\mathrm{Iy}2\right)\;-\;(\mathrm{Ix}1\;+\;\mathrm{Iy}1)}{\mathrm{Io}}\;=\;\frac{2y}{\mathrm{Ly}}$$

where Lx, Ly are the sensor dimensions (Lx = Ly = 10 mm), and Ix1, Ix2, Iy1, Iy2 are the currents obtained from PSD electrodes according to the incidence of light, and Io is total photo current expressed as follows, Io = Ix1 + Ix2 + Iy1 + Iy2 [9].

PSD generates a weak current which is proportional to the level of illumination, and the associated signal conditioning circuitry must be carefully designed to meet the challenges of low bias current, low noise, and high gain [19]. PSD may either be operated with zero bias (photovoltaic mode) or reverse bias (photoconductive mode). Application of a reverse bias, i.e., operation in the photoconductive mode, can greatly improve both the speed of response and the linearity of the devices [19]. In the photoconductive mode, a small amount of current called dark current will flow evenly when there is no illumination. 

**Figure 4:**
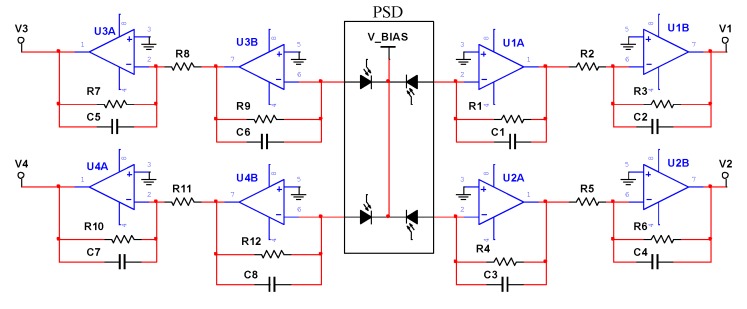
PSD signal conditioning circuit.

As shown in Figure 4, PSD signal conditioning circuit mainly contains trans-resistance amplifiers and inverting amplifiers to perform accurate current-to-voltage conversion. The PSD sensor model is S5991-01 produced by Hamamatsu. The PSD is operated in the photoconductive mode for higher switching speeds.

The Four channel output voltage signals (V1, V2, V3, V4) of PSD signal conditioning circuit are proportional to the optical current signal. Ideally, the four signal conditioning circuits have identical parameters. Therefore, the currents (Ix1, Ix2, Iy3, Iy4) can be replaced by voltage (V1, V2, V3, V4) in Equations (1) and (2) [11]. 

Ideal trans-resistance gain $A_{k}$ is Vi/Ii, for example $A_{1}$ is shown in Equation (3).

(3) $$A_{1}=-\frac{1}{R2}\cdot \frac{R1}{1+SR1C1}\cdot \frac{R3}{1+SC2R3}$$

Equation (4) shows the model of output voltage including component noise (Shot noise, Thermal noise and amplifiers noises), ADC quantization noise, etc.

(4) $$V_{k}=\mu_{k}A_{k}I_{k}+\delta_{k}$$

where k = {1,2,3,4}. $A_{k}$ is ideal trans-resistance gain, and $\mu_{k}$ is unbalanced gain coefficient, which are mainly determined by tolerance of circuit components and temperature changes. In fact, four $A_{k}$ are always different due to the asymmetry of circuit parameters. A gain equalization correction method based on the origin data is proposed to correct the factor $\mu_{k}$ for eliminating the calculation error of unbalanced gain. In addition, the other major contributor to the positioning error is $\delta_{k}$, which should be deducted when calculating coordinates.

**Figure 5:**
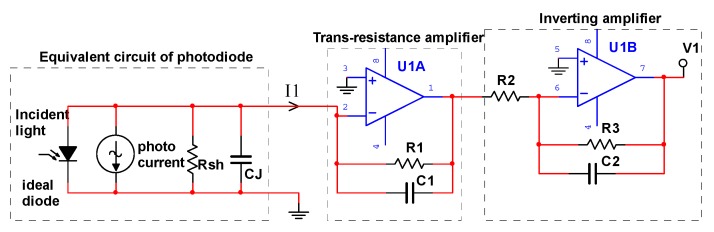
Photodiode equivalent conditioning circuit.

The equivalent circuit of channel 1 is shown in Figure 5, and the other three channel circuits are the same as channel 1. Rsh is the interelectrode resistance of PSD. Diode capacitance CJ is a function of junction area and the diode bias voltage. 

It is vital for the AC design of signal conditioning circuit to understand the circuit noise gain as a function of frequency, because the noise gain characteristics determine the stability of the circuit [20]. Noise gain (NG) calculated according to the circuit in Figure 5 is shown in Equation (5). As per Equation (5), we calculate zero frequency (fz) and pole frequency (fp) of the noise gain transfer function, as shown in Equations (6) and (7). A zero in the noise gain transfer function occurs at a frequency fz, and the pole of the transfer function occurs at a corner frequency fp.

**Figure 6:**
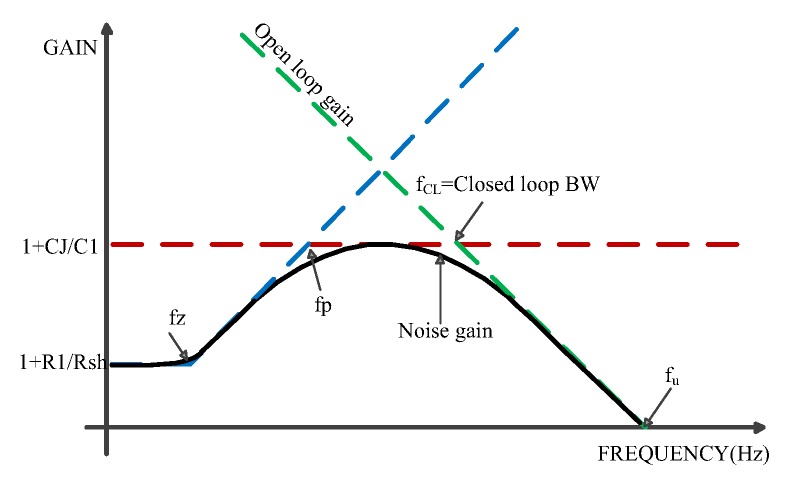
Generalized noise gain bode plot.

As shown in Figure 6, the stability of the circuit is determined by the intersecting bode diagram of the amplifier’s open-loop gain and noise gain [19]. As for unconditional stability, the noise gain curve must intersect the open loop response curve with a net slope less than 20dB per decade, and the circuit phase margin is greater than or equal to 45 degrees [21]. The noise gain shown in blue dotted line intersects the open loop gain at a net slope of 20 dB per decade, which is regarded as unstable condition. Moreover, it would occur in conditioning circuit if there were no feedback capacitor (i.e., C1 = 0). The noise gain baud diagram (black curve) represents the total noise gain (frequency range) for a given photodiode circuit design. The operational amplifier voltage noise, operational amplifier current noise, and resistance noise will increase the total system noise. However, the operational amplifier voltage noise in the first level is usually the main noise.

The open loop gain (AOL) of operational amplifier (the dotted green line) is the main factor to determine the total bandwidth of a given photodiode circuit design. A faster operational amplifier will move the AOL line to the right, while a slower operational amplifier will move the AOL line to the left. It is important to choose the right operational amplifier for this circuit design. If the operational amplifier is slow, the design will not satisfy the bandwidth and setup time specifications. If the operational amplifier is too fast, the design will generate more noise and have fewer significant digits. The AOL curve can be found in the OP amplifier data sheet.

The input impedance of the circuit caused by CJ will decrease when we increase the frequency from DC. This will increase the noise gain by feeding less output back to the operational amplifier’s inverting input. This process will continue until C1 reaches the same impedance as R1, at which point the black noise gain curve deviates from the dotted blue line. At low frequencies, the noise gain is 1 + R1/Rsh. At high frequencies, it is 1 + CJ/C1. The intersection frequency of noise gain and open-loop gain curve is called closed-loop bandwidth. The red curve determines the maximal noise gain. Moreover, it can be changed by changing parameters of the circuit.

(5) $$\mathrm{NG}=\left[1+\frac{R1}{Rsh}\right]*\frac{1+s*\left[\frac{R1*Rsh}{R1+Rsh}\right]\left(C1+Cin\right)}{1+S*R1*C1}$$

(6) $$\mathrm{fz}=\frac{1}{2\pi *\left[\frac{R1*Rsh}{R1+Rsh}\right]*\left(C1+Cin\right)}$$

(7) $$\mathrm{fp}=\frac{1}{2\pi *R1*C1}$$

## 3. Results

### 3.1. Static Output Voltage

**Figure 7:**
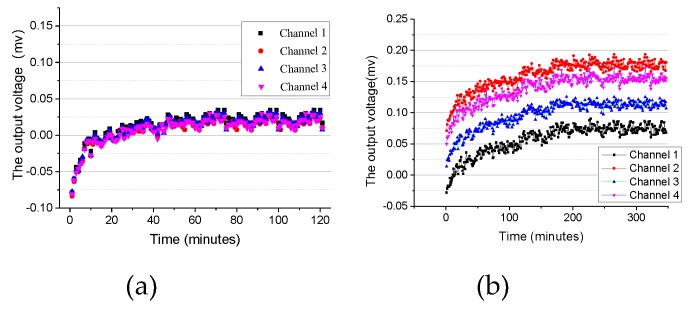

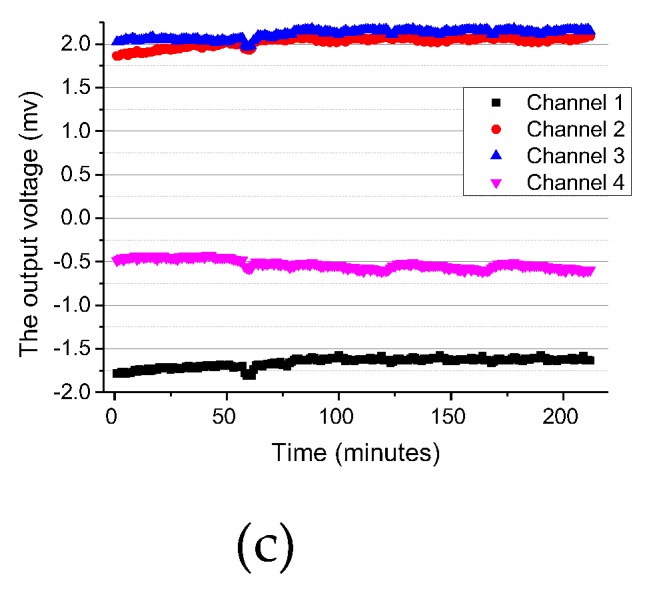
(**a**) The output voltage of A/D converter without input. (**b**) The output voltage of conditioning circuit without PSD input. (**c**) The output voltage of conditioning circuit with PSD input.

The circuit design and component parameter selection are conducted based on circuit stability analysis. We used the typical values given by the manufacturer for Hamamatsu S5991-01. The amplifier model is AD8622, with low power consumption, high precision, rail to rail output. Therefore, values of other components used were: R1 = 309 kΩ, C1 = 56 pF, R2 = 3.3 kΩ, R3 = 3.3 kΩ, C2 = 56 nF, Rsh = 10 kΩ, Cj = 500 pF [21]. To illustrate static output voltage, Table 1 shows the statistical voltages in three cases in Figure 7. Figure 7a shows the output voltage of A/D converter without any input. The AD converter has good consistency in the output of four channels, and the static output reaches 10 microvolts. Figure 7b shows the output voltage of conditioning circuit without PSD input. The output voltage of conditioning circuit without PSD input reaches 100 microvolts, which mainly comes from thermal noise caused by feedback resistance, shot noise caused by gate current of amplifier. Figure 7c shows the output voltage of conditioning circuit in a dark room. The dark current will cause the main contribution in the output voltage of conditioning circuit of this stage, which reaches 2 V. These will affect the positioning accuracy of PSD, especially dark current and the imbalance of circuit element parameters.

### 3.2. PSD Positioning Calculation and Data Analysis

**Figure 8:**
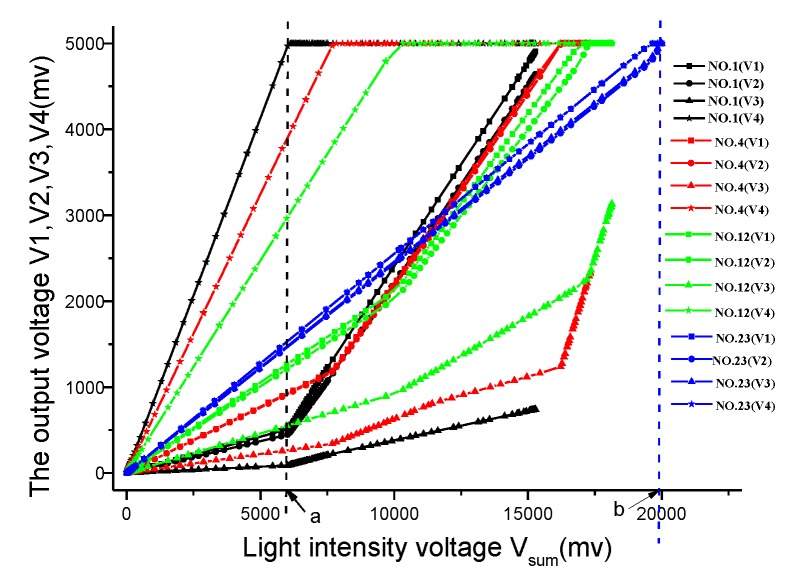
The output voltage of each channel at different PSD incident light position.

Figure 8. shows the output voltage of each channel at different PSD incident light positions which are identified as No.1, No.4, No.12, and No.23. With the increase of light intensity, the output voltage curve of the channel will turn (e.g. point a), which means that the circuit has entered a saturated state, resulting in additional calculation error of the position of the incident point. As can be seen from Figure 8, the farther away from the central point of PSD, the less light intensity is required to reach the saturation state. For example, the output voltage of channel 4 (black star curve) first reaches saturation state after point a at position No.1, which locates at the edge of the PSD. The light intensity voltage corresponding to the point a is about 6 V. The four-channel output voltage (blue curve) is in the critical saturation state when the light intensity voltage reaches 20 V. The worst position is located at the four vertices of the PSD, which is determined by the nature of the PSD. As per the calculation, the maximum saturation light intensity ratio corresponding to the central position and the edge position of PSD is about 5. This feature of PSD restricts the dynamic variation range of incident light source intensity, which is a very unfavorable condition for solar tracking.

**Figure 9:**
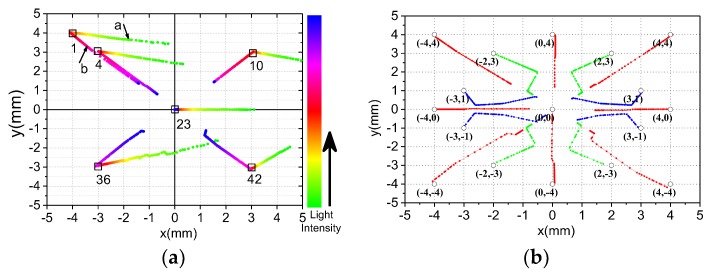
(**a**) Spot location coordinates with varying light intensity; (**b**) spot location coordinates at saturation.

**Figure 10:**
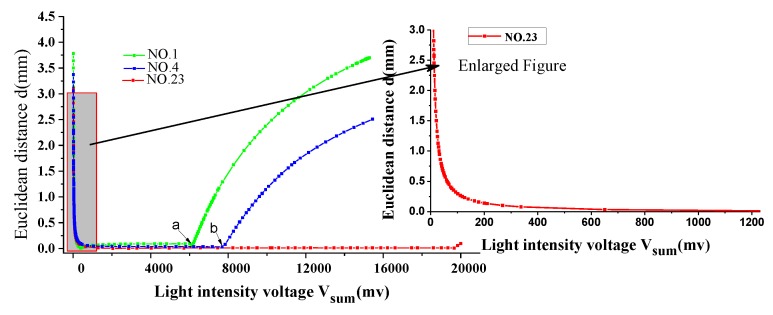
The Euclidean distance between the ideal spot position and the incidence spot position calculated according to Equations (1) and (2) with different light intensity.

Figure 9a shows the spot location coordinates with varying light intensity at different PSD incident light positions. Figure 9b shows the spot positioning coordinates with saturated light intensity at different PSD incident light positions. As can be seen intuitively from Figure 9, the calculated coordinates of the position of the spots will produce large deviations when the light is very weak or very strong. However, we found an interesting phenomenon that the calculated coordinate positions tend to the center from different directions with the saturation light intensity increasing (e.g., segment b of the coordinate curve of NO.1). It means that we can still roughly control the servo system to track the sun with poor accuracy even in the case of light saturation, but the system may exit from the saturation state when the point is close to the center. Figure 10 shows the Euclidean distance between the ideal spot position and the incidence spot position calculated by Equations (2), (3) with different light intensity. As can be seen from the enlarged image of PSD Center NO.23, the Euclidean distance error almost increases exponentially (up to 3 mm) when the light intensity voltage is below 400 mV. As shown in Figure 9a, the calculated coordinate deviates to the right (e.g., segment a of the coordinate curve of NO.1). Therefore, it is impossible to track the sun. Next, we will focus on the solution to this problem.

## 4. Adjustment Method and Experimental Analysis

The calibration method is mainly carried out in two aspects:

- Firstly, we deduct the static output voltage ($\Delta V_{i},i=\left\{1,2,3,4\right\}$).
- Secondly, we carry out equalization calibration of four channel trans-resistance gain by adjusting the gain coefficient $k_{i},\left(i=\left\{1,2,3,4\right\}\right)$.
- The calibration calculation formulas are shown in Equations (8) and (9).
  (8) $$x=\frac{\mathrm{Lx}}{2}\frac{\left(k_{2}V_{2}-\Delta V_{2}+k_{3}V_{3}-\Delta V_{3}\right)-\left(k_{1}V_{1}-\Delta V_{1}+k_{4}V_{4}-\Delta V_{4}\right)}{\sum_{j=1}^{4}(V_{j}-\Delta V_{j})}$$
  (9) $$y=\frac{\mathrm{Ly}}{2}\frac{\left(k_{2}V_{2}-\Delta V_{2}+k_{4}V_{4}-\Delta V_{4}\right)-\left(V_{1}-\Delta V_{1}+k_{3}V_{3}-\Delta V_{3}\right)}{\sum_{j=1}^{4}(V_{j}-\Delta V_{j})}$$

The four channel PSD output voltages ${\mathrm{Vd}}_{j}$ (j = 1,2,3,4) were acquired in the dark room. 

### 4.1. The Static Voltage Deduction of Signal Conditioning Circuit

The static output voltages of the conditioning circuit tested in dark room are as follows:

$${\mathrm{Vd}}_{1}=-1.665\;\mathrm{mV},{\mathrm{Vd}}_{2}=2.121\;\mathrm{mV},{\mathrm{Vd}}_{3}=2.282\;\mathrm{mV},{\mathrm{Vd}}_{4}=-0.691\;\mathrm{mV}.$$

If the static voltages measured in the dark room are used for calibration directly, we find that these values have good calibration effect only in the partial range of PSD, so we use genetic algorithm to find a set of global optimal static voltage deduction data in PSD.

Genetic algorithm parameters are as follows:

- Individual vector: $\left\{\Delta V_{1},\Delta V_{2},\Delta V_{3},\Delta V_{4}\right\}$, variable range {−2Vd_jmax_, +2Vd_jmax_}, population size: 100. where, ${\;\mathrm{Vd}}_{\mathrm{jmax}}=2.282$.
- Selection function: stochastic uniform; mutation function: adaptive feasible; crossover function: intermediate.
- Fitness function: The fitness function Equation (10) is defined as the sum of Euclidean distance errors between all calibration points and their real positions.
  (10) $$Fitness=\overset{9}{\underset{i=1}{\sum }}\overset{90}{\underset{m=1}{\sum }}\sqrt{{\left(x_{im}-x_{i}^{*}\right)}^{2}+{\left(y_{im}-y_{i}^{*}\right)}^{2}}$$
  where:${\;x}_{\mathrm{im}}$ and $y_{\mathrm{im}}$ are the coordinates of calibration points (label i = {1,2,3,20,23,26,43,44,45}) calculated by Equations (3) and (4). $\;x_{i}^{*}$ and $y_{i}^{*}$ are the real coordinates of calibration points.
- Stopping criteria: The algorithm terminates if the cumulative change of the fitness function in stall generations is less than function tolerance (1e–6).

**Figure 11:**
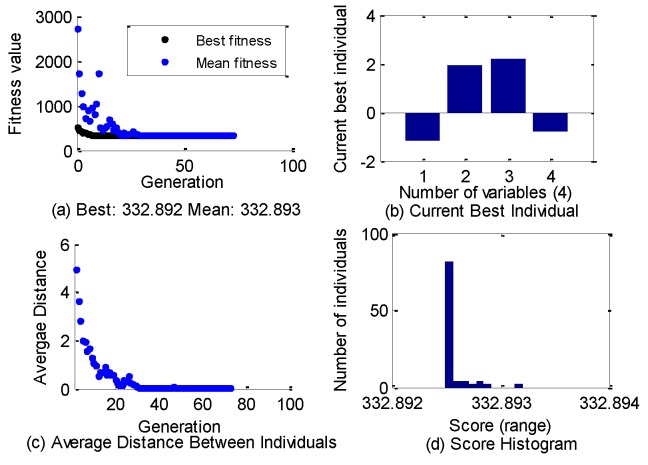
Optimize parameters curves of genetic algorithm.

**Figure 12:**
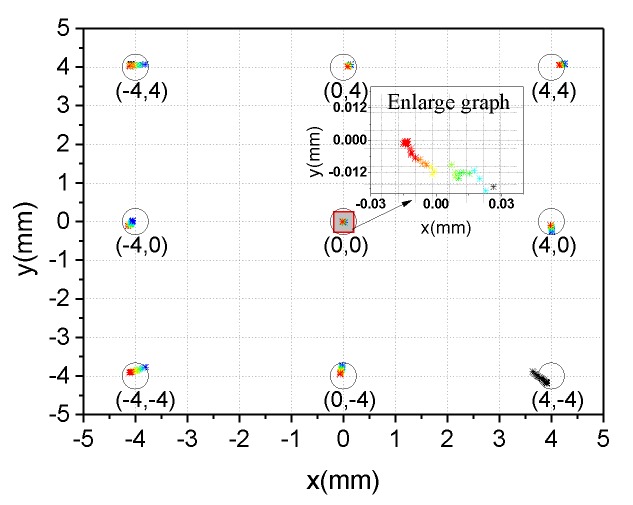
Calibration point coordinates with static voltage deduction.

The parameter curves optimized by genetic algorithm are shown in Figure 11. The optimization results of Genetic algorithm are shown as below. Objective function value is 332.892. Optimization termination condition is that average change in the fitness value is less than function tolerance(1e–6). The optimal static voltage deduction data is as follows: $\Delta V_{1}$ = −1.172, $\Delta V_{2}=1.932$, $\Delta V_{3}=2.212$, $\Delta V_{4}=-0.761$. As shown in Figure 12, the positioning coordinates of the nine calibration points converge to the ideal position. It is clear that the maximum positioning deviations of the x-axis and Y-axis of the center point are 0.03 mm and 0.024 mm, respectively. It is already a good result. Next, we will use other sampling points to verify the validity of this set of data.

### 4.2. The Equalization Calibration of Four Channel Trans-Resistance Gain

As described, unbalanced gain is mainly determined by tolerance of circuit components and temperature changes. We use the gain ratio between different channels to correct gain coefficient. The positioning error caused by temperature change can be neglected under the condition of gain balance, because the four-channel circuit are placed in the same environment and have the same temperature coefficient. 

The steps of the equalization gain calibration are as follows:

- PSD center position (NO.23) data is selected for gain calibration. Ideally, the output voltage of the four channels at NO.23 is the same when the input light intensity is different, which is determined by the characteristics of the PSD.
- The output data of the four-channel output is fitted with a straight line based on Equation (11).
- The gain ratio between different channels is calculated according to the slope of the straight line, and channel 1 is taken as a reference. The gain ratio $k_{i}$ is the ratio of the gain of channel 1 to other channels, as shown Equation (11).
- The raw output data of PSD is calibrated by gain ratio $k_{i}$, and then the position of incident light can be recalculated based on Equations (8) and (9).
  (11) $$y=G_{k}x+B_{k}$$
  (12) $$k_{i}=\frac{G_{1}}{G_{i}}$$
  where i={1,2,3,4}.

**Figure 13:**
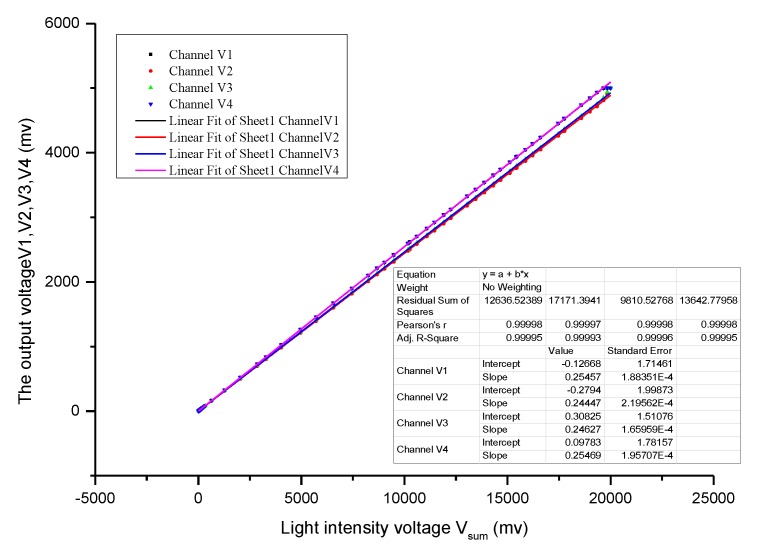
The fitting curve of 4 channel output voltages at PSD center point.

As shown in Figure 13, the data of 4 channels at the PSD center point were fitted linearly. The four fitting curves are as follows:

$$V1=0.25457V_{sum}-0.12668$$

$$V2=0.24447V_{sum}-0.2794$$

$$V3=0.24627V_{sum}+0.30825$$

$$V4=0.25469V_{sum}+0.09783$$

**Figure 14:**
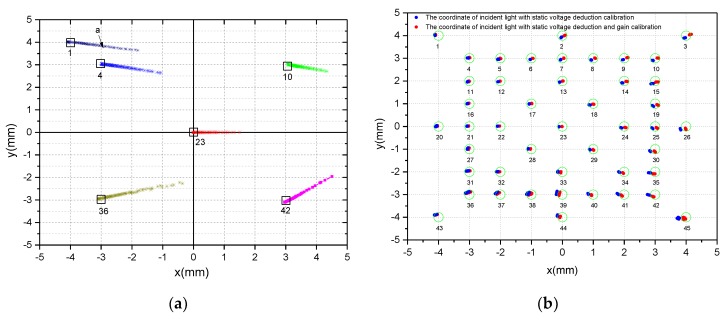
(**a**) The coordinates of sampling points without any calibration. (**b**) The blue coordinates with static voltage deduction calibration and the red coordinates with static voltage deduction and equalization gain calibration.

From the Equation (12), we calculated the gain ratio $k_{1}=1,k_{2}=1.041,{\;k}_{3}=1.033,{\;k}_{4}=0.999$. To illustrate the effectiveness of the calibration algorithm, the calculated coordinates of all 45 points are shown in Figure 14 when the total light intensity voltage of 4 channels is greater than 40mV. If the total output voltage $V_{\mathrm{sum}}$ is lower than the threshold value of 40 mV, the system will stop solar tracking. Therefore, we only care about the position coordinates of the spots where the total output voltage is greater than 40 mV. Some uncalibrated spots were selected to make an intuitive comparison, as shown in Figure 14a (label i = {1,4,10,23,36,42}). Compared with Figure 14b, we found that the coordinates of calibrated spots converge to ideal positions as far as possible. Among them, blue points coordinates are calibrated by static voltage deduction, while red points are further calibrated by unbalanced gain based on static voltage deduction. Obviously, the blue coordinates and the red coordinates have similar convergence, but the red coordinates are closer to the ideal coordinate position, especially in the central region position of PSD (e.g., NO.23, NO.17, NO.18, NO.28, NO.29).

**Figure 15:**
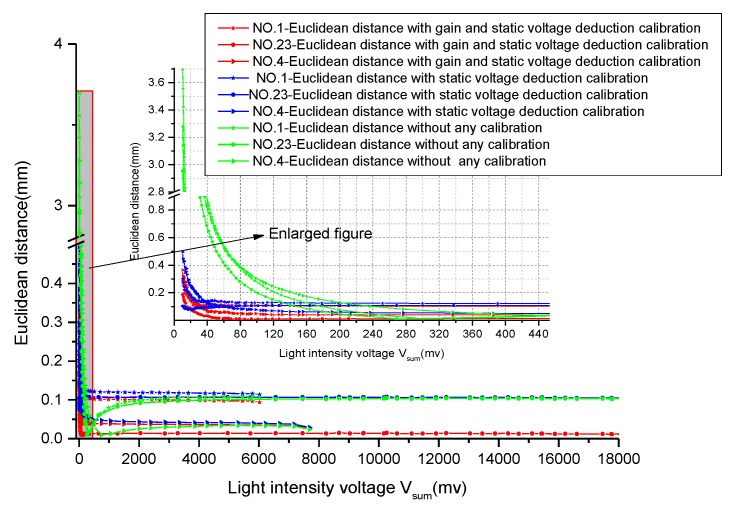
The curve of the Euclidean distance between the ideal spot position and the calculation spot position with different conditions.

As shown in Figure 15, three points (NO.1, NO.4, NO.23) are selected to calculate the Euclidean distance to illustrate the calibration effects. They represent the edge, middle, and center areas of the PSD, respectively. As shown in the local enlarged figure of Figure 15, compared with the $V_{\mathrm{sum}}\;$voltage range from 40 to saturation, the Euclidean distance after calibration is still larger in the voltage $V_{\mathrm{sum}}\;$range below 40 mV due to the influence of circuit noise and stray light. The data in Table 2 shows the average Euclidean distance and standard deviation of three coordinate points in different calibration methods, when the voltage $V_{\mathrm{sum}}\;$is between 40 mV and 400 mV. The green, blue, and red curve represents the Euclidean distance of the coordinate point without any calibration, with static voltage deduction calibration, and with static voltage deduction and equalization gain calibration, respectively. The static voltage deduction calibration makes the coordinates of the points with different light intensity converge to the ideal position, and the gain calibration further improves the positioning accuracy. Compared with uncalibrated data (a) and calibrated data (c) in table 2, the mean Euclidean distance can be reduced to 0.11Fimm at NO.1, 0.05 mm at NO.4, and 0.01 mm at NO.23. Especially in the central region, it is nearly 20 times smaller. Under the unsaturated condition, the average Euclidean distance and standard deviation of the calibration point in the range of $V_{\mathrm{sum}}$ greater than 40 mV are as follows: NO.1 (0.11 mm, 0.006), NO.4 (0.05 mm, 0.009), NO.23 (0.01 mm, 0.003). The average Euclidean distance (c) in Table 2 shows a very small deviation from the total (e.g., 0.3 um at NO.23, 0.004 mm at NO.4, 0.004 mm at NO.1). The overall precision is also very good, and the coordinates of all points almost converge to the ideal position with standard deviation 0.003 at NO.23, 0.009 at NO.4, 0.006 at NO.1. 

### 4.3. Experimental Test

**Figure 16:**
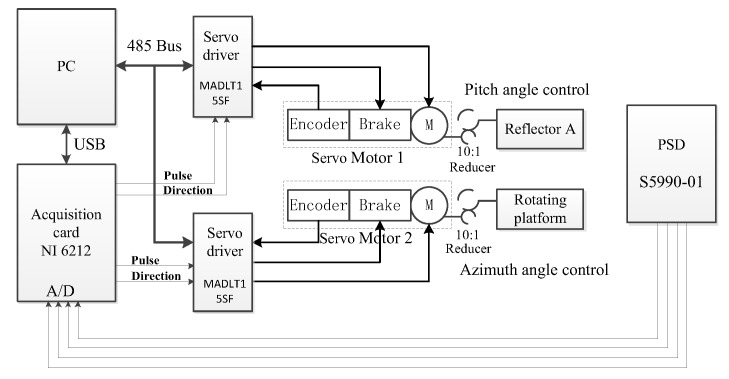
The schematic diagram of SOF-FTIR control system.

**Figure 17:**
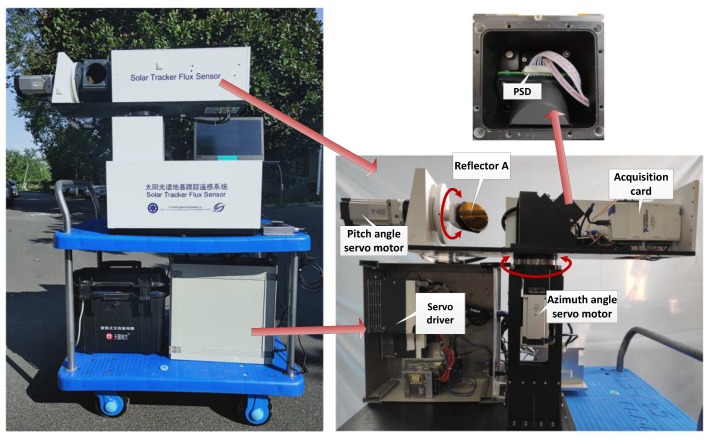
SOF-FTIR used for outdoor experimental tests.

Outdoor experimental tests were carried out to verify the accuracy of position detection of solar spot. The control system schematic diagram and the experimental apparatus are respectively shown in Figure 16 and Figure 17. The Panasonic servo system was selected for solar tracking closed-loop control. The model of servo motor 1 and 2 are respectively MSMF5AZL1B1 (50 W) and MHMF022L1B1 (200 W). The reduction gears with a reduction ratio of 10 to 1 are connected between the motor and the optical platform to improve torque and position resolution. The model of servo driver is MADLT15SF working in high resolution position control mode. The motor rotates one cycle corresponding to 10,000 control pulses. Therefore, the angle resolution of the servo control system is 0.0036° after a 10:1 reducer, which is far less than 0.2376° (Maximum divergence angle). PC sets the working parameters of servo driver through 485 bus, then collects PSD output signal through NI acquisition card (NI 6212, 16-bit, 400 KS/s) for calculation and outputs the pulse and direction control signal of servo driver after calculation.

**Figure 18:**
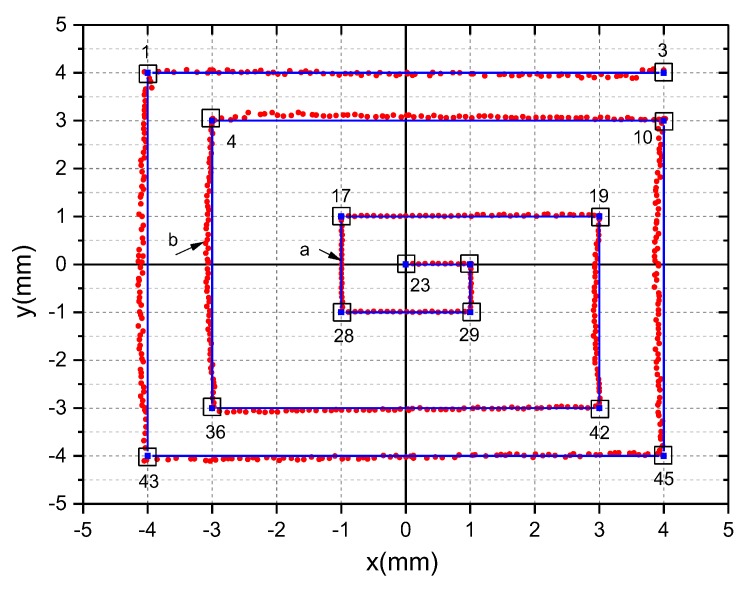
Position detection of solar spot based on SOF-FTIR.

The azimuth Angle servo motor and the pitch Angle servo motor are respectively controlled to move the solar spot on the PSD according to the preset path shown in the blue curve of Figure 18. The spot position coordinate is detected in real time, as shown in the red curve of Figure 18. In contrast, there is a slight cylindrical distortion between the detected spot trajectory and the preset path. Ideally, the angle between the Reflector A and the PSD should be 45°. However, the actual installation will always produce deviation which is the main cause of cylindrical distortion. The system vibration will cause the spot position deviation, which is main contribution of spot detection position jitter. The essence of PSD itself determines that the detection accuracy of the central region is higher than edge region. Therefore, it can be seen from figure 18 that the PSD central region has a good detection effect.

## 5. Conclusions

In this study, PSD is applied in mobile SOF-FTIR system to detect the position of solar spot and realize the closed-loop control of solar tracking. The error factors of PSD point positioning are analyzed in detail. The PSD signal conditioning circuit is designed, and its performance is evaluated by experiments. A voltage model for PSD signal conditioning circuit has been established to investigate the noise factors. The model shows that the positioning error is mainly related to PSD dark current and circuit gain. A static voltage deduction calibration method based on genetic algorithm is proposed to eliminate the effect of dark current. The gain ratio between channels is calculated based on the fitting curve slope of discrete position data of PSD center point with different light intensity for circuit gain calibration. The positioning accuracy and precision are greatly enhanced, especially when the light intensity is weak, compared with uncalibrated results. The effective total voltage detection range can be down to 40 mV (the maximum output total voltage of the central point of PSD can reach 20 V in the unsaturated state). The positioning accuracy can reach 0.01 mm at PSD central region, 0.05 mm at middle region and 0.11 mm at edge region through calibration. The positioning accuracy of the central area of PSD can reach 0.14% through calibration, the positioning accuracy of the middle area is 0.49%, the positioning accuracy of the edge area is 1.09%, and the corresponding standard deviation of the data of each area is 0.005, 0.009, and 0.014, respectively. Moreover, in the whole of the light intensity variation, the standard deviation of the experimental data curve proves that the positioning precision is consistent. As the dynamic range issue of light intensity is solved by the algorithm, the signal conditioning circuit does not need variable gain control and high trans-resistance gain, which reduces the thermal noise of gain résistance. The circuit is simple and reliable. The adjustment methods proposed in this paper improve both measurement accuracy and detection limit. The results demonstrate that the calibrated PSD positioning accuracy can meet the requirements of SOF-FTIR for solar tracking. The solar tracking experiment under mobile conditions will be carried out in the next step, which will solve the problem of tracking accuracy of servo control.

## Figures and Tables

**Table 1:** The output voltage statistics of each channel with different conditions.

	Mean Voltage (mV)			Minimum Voltage (mV)			Maximum Voltage (mV)		
	(a)	(b)	(c)	(a)	(b)	(c)	(a)	(b)	(c)
Channel1	0.013	0.057	–1.657	–0.078	–0.029	–1.808	0.034	0.091	–1.578
Channel2	0.009	0.161	2.026	–0.083	0.071	1.864	0.028	0.193	2.097
Channel3	0.011	0.097	2.114	–0.077	0.014	1.961	0.031	0.125	2.177
Channel4	0.009	0.138	–0.528	–0.081	0.049	–0.614	0.029	0.171	–0.421

**Table 2:** Comparison between different calibration: **(a)** uncalibrated data, **(b)** the data with static voltage deduction calibration, **(c)** the data with static voltage deduction and equalization gain calibration.

Spot	NO.1			NO.4			NO.23		
	(a)	(b)	(c)	(a)	(b)	(c)	(a)	(b)	(c)
Mean (mm)	0.41	0.13	0.11	0.38	0.08	0.05	0.28	0.10	0.01
Standard Deviation	0.26	0.01	0.01	0.22	0.02	0.01	0.20	0.01	0.01
